# Peritoneal tuberculosis: a benign differential diagnosis of ovarian cancer with peritoneal carcinomatosis (a case report)

**DOI:** 10.11604/pamj.2023.45.151.40327

**Published:** 2023-08-07

**Authors:** Anas Ahallat, Mohamed Al Amine El Mouden, Younes Aggouri, Said Ait Laalim

**Affiliations:** 1 Department of General Surgery, University Hospital Center, Tangier, Morocco

**Keywords:** Peritoneal tuberculosis, ovarian cancer, laparoscopy, case report

## Abstract

Peritoneal tuberculosis is a rare form of tuberculosis which gives a non-specific clinical picture which can be confused with several digestive pathologies. It can also mimic ovarian cancer at the stage of peritoneal carcinomatosis, hence the interest sometimes of a diagnostic laparoscopy which makes it possible to make the diagnosis which is confirmed by an anatomo-pathological study. This is the case of our patient who was initially diagnosed with ovarian cancer and the diagnosis was corrected in peritoneal tuberculosis after a laparoscopy.

## Introduction

Tuberculosis is a disease caused by a mycobacterium of the tuberculosis complex characterized by its contagiousness, and still represents a public health problem in developing countries. Indeed, in Morocco, in 2019, the number of cases estimated by the WHO was 35,000 new cases and the estimated number of deaths related to tuberculosis was 2,900 deaths, which means a specific mortality rate of 8.1 per 100,000 residents [1].

In 2020, the number of cases recorded was 29,018 cases, all forms combined, with two hundred and forty cases coinfected with tuberculosis and human immunodeficiency virus (HIV) [2]. Of which one of the most particular forms of extra-pulmonary manifestation is peritoneal tuberculosis which affects the intestine, liver, spleen, female genital tract, omentum, parietal and visceral peritoneum which accounts 1-2% of all forms of tuberculosis [3]. Taking into account that this type of peritoneal manifestation of tuberculosis disease is rare, and with a non-specific clinical presentation essentially abdominal distension, ascites, tenderness, fever and weight loss, this explains the diagnostic delay which is around 4 months, however in the female population, the presence of ascites, adnexal mass and elevated CA125 may suggest ovarian cancer, but the diagnosis of peritoneal tuberculosis which is a benign pathology occurs at a young age between the ages of 20 and 40 should not be omitted [4], while ovarian cancer occurs in an older population. The present work has set itself the objective of reporting the case of a 27-year-old woman with several tuberculous peritoneal and grelic nodules initially misdiagnosed as ovarian cancer at the stage of peritoneal carcinomatosis.

## Patient and observation

**Patient information:** a 27-year-old woman, G1P1 (G=gravidity=1, P=parity=1) referred to the emergency department for abdominal pain localized at the hypogastric level, with hemodynamic stability, without any notion of contracture or abdominal defense, with a reported notion of fever which couldn´t be found on the general examination.

**Timeline:** 1 week.

**Clinical findings:** however, on clinical examination, the presence of abdominal ascites was noted, which prompted to realize an abdominal ultrasound that confirmed the presence of abdominal ascites of moderate abundance, in addition to an ovarian mass measured at 56x48mm.

**Diagnostic assessment:** biologically the patient presented an increased CA 125 to 198 u/ml with a level of normal white blood cells at 6000 and hemoglobin at 10.4 g/dl. An additional CT scan was in favor of a possible malignant ovarian mass with peritoneal carcinomatosis, with normal chest images.

**Figure 1:**
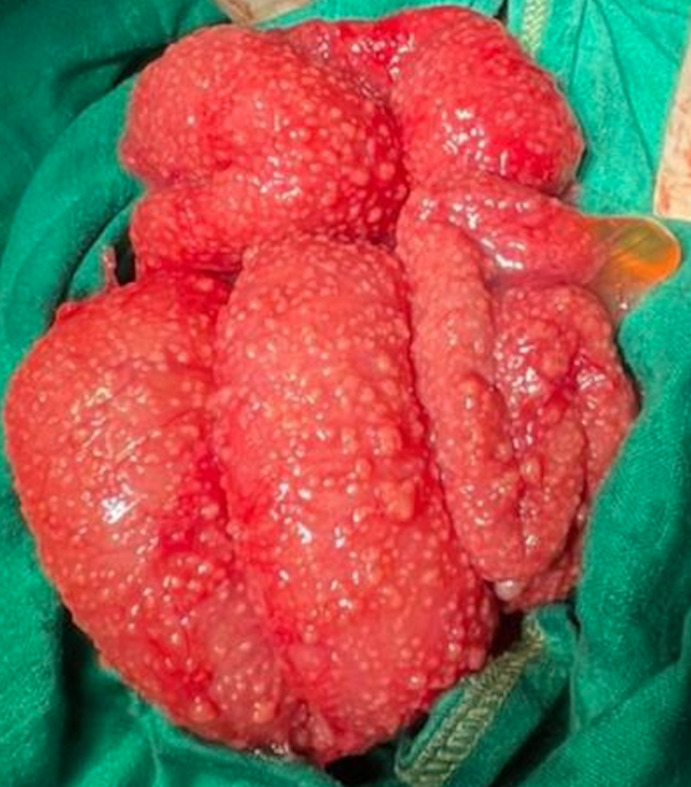
granulations of visceral peritoneum of the small intestine

**Figure 2:**
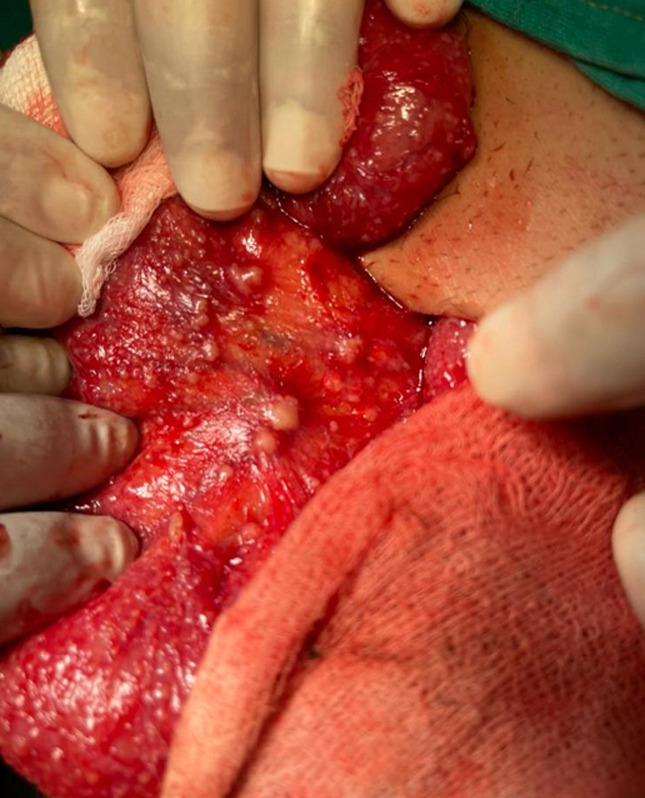
mesenteric lymphadenopathy

**Therapeutic intervention:** subsequently, the patient underwent a mini-laparotomy, finding no ovarian mass but an increase in the volume of the ovarian tubes as well as several peritoneal and small bowel deposits (Figure 1) and mesenteric lymphadenopathy (Figure 2). The anatomopathological examination of the various epiploic peritoneal samples and nodules was in favor of peritoneal tuberculosis, without the presence of cells suspected of malignancy.

**Follow-up and outcomes:** the patient was subsequently put on anti-tuberculosis treatment with good clinical improvement.

**Patient perspective:** the patient was satisfied with the treatment and happy to preserve her ovary.

**Informed consent:** the patient gave informed consent.

## Discussion

It is not always easy to distinguish between peritoneal tuberculosis and ovarian cancer at the stage of peritoneal carcinomatosis even based on the CA 125 rate, which can lead to abusive laparotomies in young patients, however, most cases can be diagnosed using a laparoscopy which seems to be the best alternative [5], especially when we know that the tumor marker, laboratory investigations and radiological imaging are also non-specific [6] because CA-125 is a glycoprotein, which is expressed by cells lining the uterine endometrium and serum levels are elevated in conditions such as ovarian malignancy, endometriosis and pelvic inflammatory disease [7]. CA-125 is also expressed by cells lining the pleura, pericardium, and peritoneum and therefore serum levels may be elevated in tuberculous peritonitis, intestinal malignancies, and in postoperative cases [7].

In our case, the clinical presentation could hardly suggest peritoneal tuberculosis, apart from the fact that the age of the patient is in favor of tuberculosis and the epidemiological context of Morocco, since it is a region of the world where tuberculosis is still rampant. Moreover, an Indian study of 26 patients who underwent laparotomy for ovarian cancer had abdominal-pelvic tuberculosis confirmed after laparoscopy and histopathology test [8]. Therefore to improve preoperative detection of tuberculosis, ascetic fluid adenosine (ADA) and PCR analysis have proven to be useful [9] and could avoid unnecessary or even dangerous laparotomy. Also, PET-CT may be a good diagnostic method, and the cost is not very high; however, further studies using PET-CT should be conducted to confirm this finding [10].

## Conclusion

In endemic areas, peritoneal tuberculosis should always be considered as a differential diagnosis while encountering a case of a young woman presenting abdominal pain with pelvic mass and ascites even with elevated rate of CA 125, because peritoneal tuberculosis can mimic malignancy and cause a delay in diagnosis. Also, when noninvasive procedures do not lead to diagnosis, invasive methods should be considered to distinguish between the two pathologies.
